# Case Report: A Giant Left-Ventricular Intramural Pseudoaneurysm Arise From Ruptured Left Sinus of Valsalva Aneurysm

**DOI:** 10.3389/fcvm.2021.753627

**Published:** 2021-12-09

**Authors:** Minghui Hua, Yufan Gao, Jianhui Li, Fang Tong, Ximing Li, Hong Zhang

**Affiliations:** ^1^Department of Radiology, Tianjin Chest Hospital, Tianjin, China; ^2^Academy of Medical Engineering and Translational Medicine, Tianjin University, Tianjin, China; ^3^Department of Physiology and Biochemistry, School of Fundamental Medicine, Shanghai University of Medicine and Health Sciences, Shanghai, China; ^4^Department of Cardiology, Tianjin Chest Hospital, Tianjin, China; ^5^School of Clinical Medicine, Tianjin Medical University, Tianjin, China; ^6^School of Clinical Medicine, Tianjin University, Tianjin, China

**Keywords:** sinus of Valsalva aneurysm, ventricular intramural pseudoaneurysm, echocardiogram, computed tomography, cardiac magnetic resonance

## Abstract

In this report, we present a case study of an extremely rare left sinus Valsalva aneurysm (SVA) rupture into the left-ventricular myocardium. Acute ozone inhalation and long-term hypertension are possible contributors to the condition. Utilizing multimodal cardiovascular imaging techniques [echocardiogram, computed tomography (CT), and cardiac magnetic resonance (CMR)], a large, left-ventricular, intramural pseudoaneurysm (IPA) arising from the ruptured left SVA, was clearly observed anatomically and functionally. Subsequently, our patient underwent patch repair and valvoplasty which offered an excellent prognosis. This report describes the manifestation of the ruptured left SVA and its possible etiology. This case also emphasizes the need for multimodal imaging for subsequent surgical repair.

## Introduction

SVA is a rare cardiovascular disease typically originating from the right coronary sinus or non-coronary sinus, and rarely from the left coronary sinus (1). Previous reports suggest that SVA pathology could involve congenital defects, previous infection, trauma, or degenerative conditions (1). Clinically speaking, SVA can present asymptomatically in patients. However, the situation becomes life-threatening in the event of a ruptured SVA. In this case report, we detail an extremely rare case of the left SVA which ruptured into the left ventricular myocardium.

## Case Presentation

A 46-year-old male patient was admitted to the hospital with intermittent back pain and chest tightness for 2 weeks. The patient works in ozone disinfection. Prior to symptom onset, he had a history of acute ozone inhalation. He recalled smelling something more pungent than usual for several days. Before coming to our hospital, he had not undergone any treatment. In addition, he denied any history of chest trauma. He had suffered from hypertension for over a decade and was treated with oral nifedipine and metoprolol. However, medication poorly controlled his blood pressure. The highest recorded systolic blood pressure with treatment was 180 mmHg. His blood pressure upon admission was 148/91 mmHg.

**Figure 1:**
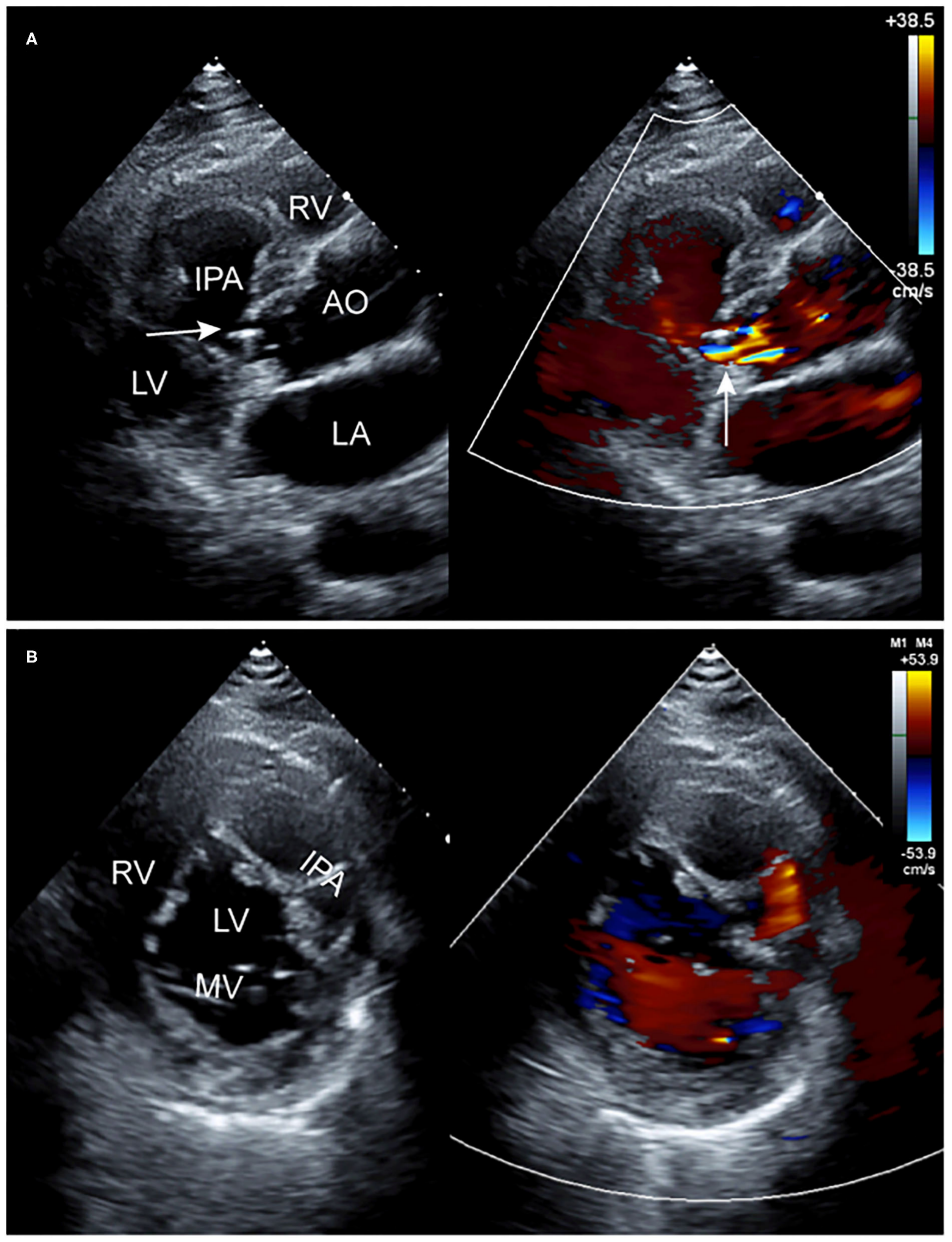
Two-dimensional transthoracic echocardiographic images. **(A)** Long-axis view of the left ventricle showing an echo-lucent cavity in the left-ventricular myocardium with abnormal blood flow on color Doppler (arrow). **(B)** Short-axis view at the MV (mitral valve) level also showing the echo-lucent cavity with partitions in the left-ventricular myocardium and abnormal blood flow on color Doppler. AO, aorta; LA, left atrium; LV, left ventricle; RA, right atrium; RV, right ventricle; RVOT, right ventricular outflow tract.

**Figure 2:**
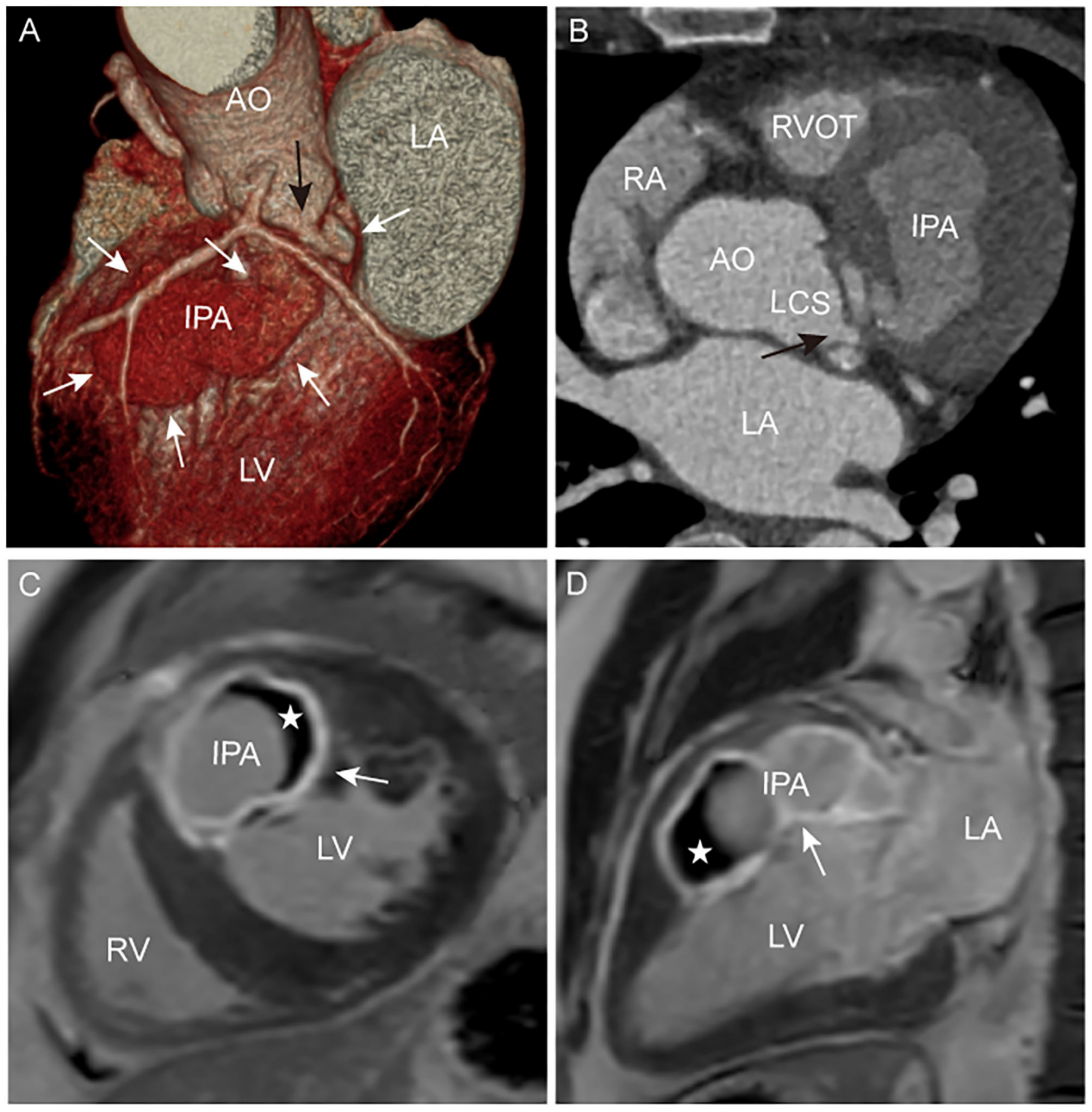
**(A,B)** CT angiogram images showing the anatomical profile of IPA (white arrow) and perforation (black arrow). **(C,D)** CMR LGE images showing the high signal intensity of the IPA with a distal low signal intensity (✰), and a linear high signal intensity (white arrow) of the IPA wall and partitions. LCS, left coronary sinus.

An echocardiogram revealed a left SVA that ruptured into the left-ventricular myocardium, forming an echo-lucent cavity (Figure 1). The left-ventricular wall had thickened resulting in uncoordinated motion and reduced systolic function. Moreover, moderate eccentric aortic regurgitation was also noted. Furthermore, CT angiograms better captured a large, left-ventricular, IPA arising from a small perforation in the left SVA (Figures 2A,B). The adjacent left ventricle and interventricular septum were compressed. With CMR examination, late gadolinium enhancement (LGE) clearly demonstrated the left-ventricular IPA with distal thrombus and a linear enhancement of the IPA wall, compatible with myocardial fibrosis (Figures 2C,D).

**Figure 3:**
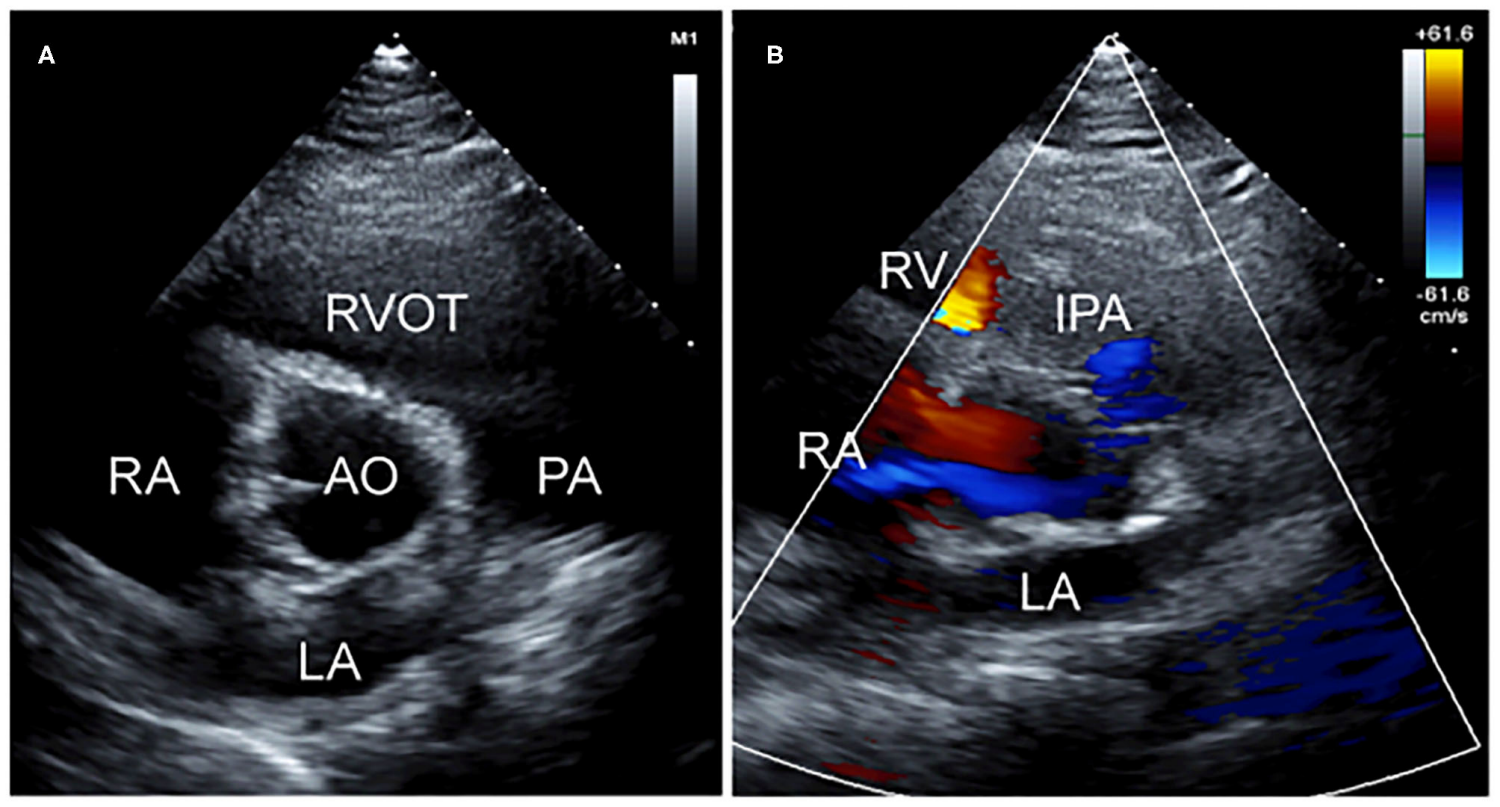
Post-operation two-dimensional transthoracic echocardiographic images. **(A)** Short-axis view at the aortic valve level showing the perforation of the left sinus of Valsalva repair and **(B)** non-standard short-axis view showing the cessation of abnormal blood flow on color Doppler.

Subsequently, the patient underwent surgery where the perforation was sutured and repaired, and the left aortic valve was lengthened with pericardial patches. Three weeks after surgery, a follow-up echocardiogram demonstrated the cessation of the abnormal blood flow in the left sinus of Valsalva (Figure 3).

## Discussion

The potentially lethal rupture of left SVA into the left-ventricular myocardium has not been commonly reported. Bricker et al. reviewed 529 SVA cases and revealed that all the SVA in this cohort ruptured into the RV, RA, or RVOT. None ruptured in the left-ventricular myocardium (1). Furthermore, in a transthoracic echocardiography study, Gu et al. reported that most of the dissections of the interventricular septum commenced in the right sinus of Valsalva. Again, none occurred in the left sinus of Valsalva (2). One report presented a case of left sinus of Valsalva which ruptured into the left-ventricular myocardium caused by congenital gene variants (3). Congenital sinus of Valsalva aneurysms may be associated with Marfan and Ehlers-Danlos syndromes (4, 5), or other structural cardiac anomalies (6, 7), such as ventricular septal defect, bicuspid aortic valve, and pulmonary stenosis. In this report, our patient lacked any familial genetic diseases associated with SVA. Indeed, multimodal cardiovascular imaging of the patient demonstrated the left SVA and ventricular IPA, without structural cardiac anomalies associated with SVA.

Our patient did have a history of ozone inhalation. To the best of our knowledge, ozone is a highly reactive and oxidizing gas. Many epidemiological investigations (8, 9) as well as animal experiments (10, 11) have revealed that ozone exposure can cause a variety of cardiovascular system impairments. These studies suggested potentially complex mechanisms to explain the cardiovascular injuries, including: increased oxidative stress, the activation of platelet and systemic inflammatory responses, increased blood pressure, and the modification of endothelial function and vascular vasomotricity. Our patient suffered from long-term hypertension that had not been well-controlled. This may have led to impairment of the vascular structure. Moreover, as the left sinus of Valsalva does not arise from the bulbar septum, it is less frequently affected by congenital lesions (7). Although it is impossible to define the precise vascular pathology in this case, we hypothesize that the etiology of the ruptured SVA may have been acute ozone inhalation and long-term hypertension.

For a ruptured SVA, patch repair is the most common and effective surgical treatment option. Our patient underwent patch repair and valvoplasty without any periprocedural complication. Alternatively, percutaneous transcatheter closure has been reported as a less invasive surgical option for selected patients.

Although SVA is rare in medical practice, it can be fatal if the vessel is ruptured. Therefore, we must emphasize the radiological method in the accurate diagnosis of SVA. Multimodal cardiovascular imaging including echocardiography, CT, and CMR is proposed. Taken together, these can reveal very detailed information of SVAs both anatomically and functionally. Patch repair, as a primary surgical option, has resulted in an excellent prognosis for our patient. In addition, this case also suggests that ozone inhalation and long-term hypertension are causative factors for SVA rupture into the left-ventricular myocardium.

## Data Availability Statement

The original contributions presented in the study are included in the article/supplementary material, further inquiries can be directed to the corresponding author/s.

## Ethics Statement

The studies involving human participants were reviewed and approved by the Ethics Committees of Tianjin Chest Hospital. The patients/participants provided their written informed consent to participate in this study. Written informed consent was obtained from the individual(s) for the publication of any potentially identifiable images or data included in this article.

## Author Contributions

MH, XL, and HZ: conceptualization. MH, YG, and JL: data collection and analysis. MH: writing. HZ: funding acquisition. FT, XL, and HZ: writing-review and editing. All authors approved the manuscript for publication.

## Funding

This work was supported by the Program of Tianjin Science and Technology Plan (18ZXZNSY00400) and Tianjin Health Science and Technology Project (MS20015).

## Conflict of Interest

The authors declare that the research was conducted in the absence of any commercial or financial relationships that could be construed as a potential conflict of interest.

## Publisher's Note

All claims expressed in this article are solely those of the authors and do not necessarily represent those of their affiliated organizations, or those of the publisher, the editors and the reviewers. Any product that may be evaluated in this article, or claim that may be made by its manufacturer, is not guaranteed or endorsed by the publisher.
